# Drug Making and Drug Taking

**Published:** 1894-04-14

**Authors:** 


### The First Century of English Medical Literature.

### I.—DRUG MAKING AND DRUG TAKING.

Some knowledge of medicine was a far more usual accomplishment three centuries ago than in the present day. Dr. Stubbs has noted "that two of the most exclusive and 'professional' of modern professions were not in the Middle Ages professions at all. Every man was to some extent a soldier, and every man was to some extent a lawyer." He might have added that every woman was to some extent a doctor, for of doctors as of lawyers there was only "a very small and somewhat dignified number." For dwellers in the country and even in many towns regular medical aid was rarely procurable. Amateur medicine, especially after the suppression of the monasteries, was not merely a fashionable pursuit, but a necessity. It was the recognised duty in country places for the great lady at the castle or hall to practice simple methods of surgery on the labourers of the estate, and administer remarkable potions to those in need. These were charitable offices which became ladies of the highest rank, and were popular among all grades of society. A vast amount of physic, too, was considered necessary to the health of every member of the household, and the "virtuous woman" had draughts suitable for all constitutions in her store-room, and was learned in the proper times and seasons for administering them. Instruction in the art of stanching wounds and letting blood, and in the properties of herbs, formed a part of the education of every well-brought-up girl; the process of distilling "waters" and preparing concoctions of the most complicated nature was taught side by side with the mysteries of baking and brewing. Medicine and cookery were, in fact, allied sciences, and in ancient cookery-books recipes for plague water may be found interspersed with directions for preparing the most savoury delicacies. In all these matters, both of physic and cooking, tradition played a most important part. Recipes were jealously guarded, and often descended for generations from mother to daughter without the secret transpiring.

In towns the number of irregular practitioners was very great. An Italian writer, who spent some years in London at the opening of the seventeenth century, comments in particular on the extraordinary number of women doctors at that period. But every household had its own infallible mixture, pill or electuary, and the people studied with deep interest for themselves the newest theories about health and disease. The origin of these multifarious traditional prescriptions is very obscure. Many probably date from the time of the Crusades, and were indirectly borrowed from the East. Others are based on the properties of herbs, then far better understood even by the uneducated than now. Others, again, seem to have sprung from a restless imagination, working on fancied analogies and unrestrained by knowledge or responsibilty.

Small as was the reading public, a very extensive demand was early created for works of a medical character. Among all classes existed a passion for self-doctoring, arising not merely from the scarceness of duly qualified practitioners, but also from the extortionate fees and arrogant bearing of most members of the profession, combined with their clumsy and cruel methods and continued want of success. Few works throw more light on the customs, beliefs, and general education of the people than the hand-books of domestic medicine, by aid of which they practised on their health and that of their servants and children, and according to whose precepts they framed their way of life. Eagerly studied, and freely annotated, in the house of courtier, country squire, and citizen, these dingy volumes represent the high water mark of scientific achievement and sanitary progress in the golden age of English literature and enterprise.

A large proportion of these works are translations from French, Italian, German, and Dutch anthors. They are done into quaint Elizabethan English, often by some eminent surgeon, who supplements his author with observations of his own, as for instance, Queen Elizabeth's surgeon edited the works of Gesner, or by some enterprising quack. Some are of purely English origin, and these of course throw most light on the state of medical knowledge in the period. They are commonly printed in Black Letter, and the well-worn pages are in many cases freely sprinkled with half obliterated manuscript notes. Here is one—a treatise described as "A most excellent homish apothecarye or homely physicke book for all the grefes and diseases of the body"—with jottings from the pen of no less a person than Spenser's intimate friend and fellow student, Gabriel Harvey. Rarely more than two or three copies—sometimes but one—have stood the wear and tear of centuries, and the precious survivor has usually attained to the dignity of a "case book," preserved under glass in solemn seclusion, and only to be handled by the curious under the safeguard of due precautions.

As one pores over the unfamiliar lettering and studies to decipher the cramped writing in the margin, what echoes of old days seem to rise from between the worn and yellow leaves. What risings while it was yet night and stir of reluctant handmaids for the brewing of these wondrous drinks! What pounding of queer substances and search after rare rubbish! What tedious observances, prohibitions, and precautions, enforced on the young and evaded by the daring! What never-ending wrangling, what acrid dogmatism over the discrepancies of rival authorities! What needless suffering! What tragic blunders! Three centuries hence will some curious inquirer disinter a volume of The Hospital and read with just such a pitying smile of superior wisdom the story of our own latest scientific developments in Medical Progress and Hospital Clinics?

